# COVID-19 Containment Efforts of a Low-Resource Nation: The First Four Months in Nepal

**DOI:** 10.7759/cureus.8946

**Published:** 2020-07-01

**Authors:** Sangita Basnet, Sameena Koirala, Basu Pandey, Janak Koirala

**Affiliations:** 1 Pediatric Critical Care, Southern Illinois University School of Medicine, Springfield, USA; 2 Pediatric Critical Care, Patan Academy of Health Sciences, Kathmandu, NPL; 3 Biology, Grinnell College, Grinnell, USA; 4 Epidemiology and Disease Control Division, Ministry of Health and Population, Kathmandu, NPL; 5 Infectious Diseases, Southern Illinois University School of Medicine, Springfield, USA

**Keywords:** covid-19, nepal, sars-cov-2, nepal covid-19, covid-19 prevention, lock-down, sars-cov-2 testing, covid-19 isolation, coronavirus quarantine, coronavirus

## Abstract

A novel coronavirus (severe acute respiratory syndrome coronavirus 2 or SARS-CoV-2) was identified in hospitalized patients in Wuhan, China, in December 2019. It rapidly spread across the globe within the span of a few months. Nepal is a low-resource country with limited critical care delivery infrastructure. Coronavirus 2019 (COVID-19), the disease caused by the virus, could potentially cause a medical catastrophe in Nepal. We reviewed all pertinent documents published in the public domain by the Ministry of Health and Population of Nepal and other relevant literature. We aimed to describe the key strategies Nepal embraced in the first four months in its attempt to curtail the disease immediately following the identification of its first case and the challenges it faced. In our review, we determined that the key steps taken by Nepal included border control to prevent the importation of cases, strict quarantine in facilities for anyone entering the country, early case detection, and isolation of all infected cases irrespective of symptoms. Testing capabilities, quarantine facilities, and isolation beds were also rapidly increased. We discuss how Nepal achieved some success in the first four months between January 13, 2020, when the first case was identified, to May 13, 2020. However, it faced several challenges that ultimately led to an exponential rise in cases thereafter.

## Introduction and background

In December 2019, a novel coronavirus, subsequently termed severe acute respiratory syndrome coronavirus 2 (SARS-CoV-2), was identified in hospitalized patients in Wuhan, China [1]. Coronavirus disease 2019 (COVID-19), the disease caused by the virus, was declared a pandemic by the World Health Organization (WHO) on March 11, 2020 [2].

Nepal is a small mountainous nation in Asia situated between its much larger neighbors, India and China. With 39% of its 28 million population living below the $3.20 per person per day international poverty line, and a gross national income of $970 per capita, it is one of the poorest countries in the world [3]. China borders Nepal on its north and has extensive sociopolitical relations with the Himalayan nation. At the time COVID-19 emerged, there were close to 200 Nepali students in Wuhan, the epicenter of the disease [4]. The first case of COVID-19 was a 32-year-old Nepali man, returning from Wuhan University of Technology, who presented to Sukraraj Tropical and Infectious Disease Hospital in Kathmandu, the capital city of Nepal, on January 13, 2020 [5]. He was diagnosed by a nasopharyngeal swab sent to Hong Kong prior to the availability of testing in Nepal.

By May 13, four months after the first case was diagnosed, 245 infected cases were identified, with zero deaths. The United Nations had estimated 1500 infected cases in Nepal in the first month alone based on a comparison of the ratio of the population and patients of Nepal with those in China [6]. Considering the limitations of the healthcare infrastructure in this low-resource country and particularly a lack of critical care capacity [7-8], Nepal opted for the strategy of early deflection of COVID-19. We examine the steps that were taken between January 13, when the first infected case was identified, to May 13, four months later, in an attempt to avert a major medical disaster.

## Review

Preparation for the identification and management of COVID 19 (January 28, 2020, to March 11, 2020)

Once the first case was identified, Nepal started preparing for the epidemic, focusing mainly on the identification and management of cases [9]. A High-Level Coordination Committee under the chairmanship of the Prime Minister and Minister of Defense was formed for oversight of preparation and response activities. Five hub hospitals and 13 satellite hospitals were designated COVID-19 hospitals, requiring dedicated space for the isolation of infected individuals. Expert teams were formed to formulate guidelines for the treatment, testing, and management of COVID-19. Ongoing communication was established among the Central and Provincial Health Emergency Operation Centers and the Ministry of Health. Temperature monitoring was instituted at the Tribhuvan International Airport, the only international airport in Nepal.

On March 11, 2020, mandatory self-quarantine of all individuals arriving from the eight nations (China, Italy, Spain, Iran, South Korea, Germany, France, and Japan) that had community spread was initiated. Health screening consisting of a questionnaire for symptoms and a temperature check was instituted at 43 Points of Entry (PoE) from neighboring nations, India and China.

Strategies to prevent the entry of the disease (March 12, 2020, to March 21, 2020)

The High-Level Committee on COVID-19 in Kathmandu decided on March 12 to make every attempt to prevent the entry of the virus into Nepal [9]. On March 14, all entry visas were suspended and all land PoE shut down [10]. Passengers that had arrived in Nepal were requested to stay in self-quarantine and report to the Sukraraj Tropical and Infectious Disease Hospital in case of symptoms [9].

Surveillance and containment efforts (March 24, 2020, to May 13, 2020)

Lockdown

The second case, a 19-year-old who had arrived in Kathmandu from France, tested positive for SARS-CoV-2 on March 22, 2020. This led to the decision to lock down the nation on March 24. Only essential services, including pharmacies and grocery stores, could open. Citizens could only leave their houses at designated time periods [9]. All domestic and international flights were halted. Maintaining physical distancing, masks, hand washing, and hand sanitizers were encouraged.

Towards the end of March, there were five cases that had arrived from China, Europe, and Dubai that tested positive and were placed in isolation in COVID-19-designated hospitals in Kathmandu. Trained personnel under the Epidemiology and Disease Control Division (EDCD) were mobilized to conduct extensive contact tracing based on their flight details and movement history to identify individuals with a potential infection. A team was even mobilized to a village outside the capital city to investigate contacts of a case in Kathmandu [9].

Terrified villagers enforced lockdown and quarantine, imposing their own rules utilizing age-old “Mukhiya” (village chief) traditions, at times even preventing their own relatives working in neighboring towns from entering their villages by barricading entry points [11].

This state of lockdown continued for almost three months until mid-June. Lockdown/quarantine measures were strictly reinforced by the security sector (police, border management, corrections). Police presence was expansive and powerful. They implemented cash fines, confiscation of vehicles, and even imprisonment for failure to adhere to quarantine measures.

Improving and Expanding Health Care Resources

The number of hospitals for the management of COVID-19 was increased to 20 designated hospitals, 25 hub hospitals, and 66 provincial hospitals [12]. Isolation beds were created rapidly in all seven provinces. By May 13, isolation and quarantine beds were increased to 3198 and 51,539, respectively [9]. An attempt was made to strengthen intensive care units and add additional ventilators. By the end of March, however, there were just 1029 ICU beds with 552 ventilators nation-wide, and over half were situated within the capital city [9]. Various private and public organizations aided in the training of health care workers and provided them with gowns, gloves, masks, and eye protection. The scarcity of personal protective equipment (PPE), particularly N95 masks and gowns, has been an ongoing concern.

The High-Level Coordination Committee formed a COVID-19 Crisis Management Committee (CCMC) chaired by the deputy prime minister and defense minister to monitor, coordinate, and manage all COVID-19 prevention, control, and treatment activities. Furthermore, to make this more effective, District level Crisis Management Centers were also established.

COVID-12 Laboratory Facilities

**Figure 1:**
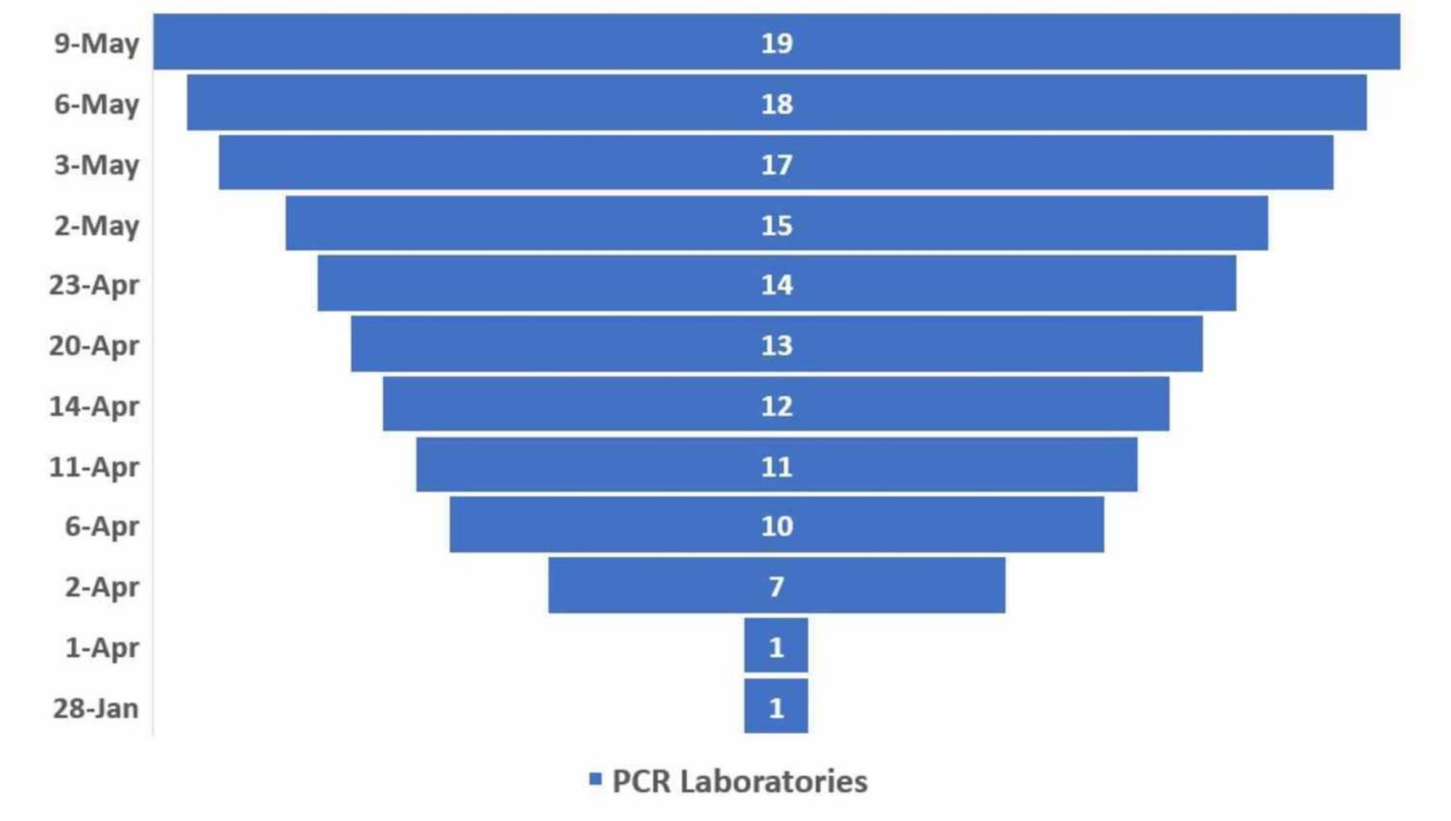
Increase in laboratories offering RT-PCR assays RT-PCR: reverse transcription-polymerase chain reaction

The testing capability was scaled up rapidly (Figure 1) [9]. By May 13, around 2000 reverse transcription-polymerase chain reaction (RT-PCR) assays were being done every day. Early February, an RT-PCR primer for SARS-CoV-2 was made available at the National Public Health Laboratory (NPHL) in Kathmandu. By May 13, there were 19 such laboratories providing services around the nation. Since these were newly set-up facilities consisting of personnel with limited experience, a nine-member expert team was formed to validate the tests. Furthermore, samples of all presumptive cases were cross-verified at NPHL before the final diagnosis. A protocol for the establishment of an RT-PCR laboratory was set forth, requiring at least a masters-level microbiologist with some experience in molecular microbiology.

Limiting Importations, Hotspots, Identification of Cases, and Contact Tracing

Even though all flight services were suspended, Nepal remained vulnerable to the transport of the virus from its two neighboring nations, China and India. High mountains separate Nepal from China on the north, hence, there are limited PoE that have remained closed since late January 2020. However, the extensive open border to the south with India is easily accessible through several official and unofficial ground-crossing PoE.

To prevent the importation of the disease, starting April 23, “Import Nirdeshika” (protocol) was initiated at all PoE [9]. Accordingly, a certificate of disinfection for transport vehicles, a self-declaration form, and a health check of all personnel were required at the health desk. Vehicles were kept in the holding yard for disinfection. Any suspicion prompted the individuals to be escorted to designated isolation/quarantine centers.

Mapping of migrant and vulnerable populations was conducted by Nepal Army and personnel trained and deployed by the EDCD. Hotspots were determined based on PoE into Nepal and reported by vigilant citizens, border control, security forces, healthcare workers, and, recently, by mobile tracking. On April 24, a decision was made to form a three-member case investigation and contact tracing teams (CICTTs), consisting of a public health professional, laboratory technician/assistant, and paramedic/nurse, utilizing local manpower to expedite and simplify screening and testing. Accordingly, in early May, 1012 such teams were trained by the EDCD and mobilized [9].

Quarantine, Test, Identify, Isolate

All international travelers into Nepal, via air or ground, and those who did not have feasible home-based quarantine facilities or were violating it were kept in quarantine centers for a minimum of 14 days [9,13]. The WHO advised that all confirmed cases, even mild cases, should be isolated in health facilities, to prevent transmission and provide adequate care [14]. Accordingly, both confirmed and suspect cases were kept under strict isolation in designated COVID-19 hospitals. Guidelines were created to monitor quarantine management by the government of Nepal. Food and daily necessary commodities were provided by the government. By May 13th, 15,600 individuals were in quarantine in facilities around the nation, including schools, tents set up in open fields, other large public buildings and arenas.

**Figure 2:**
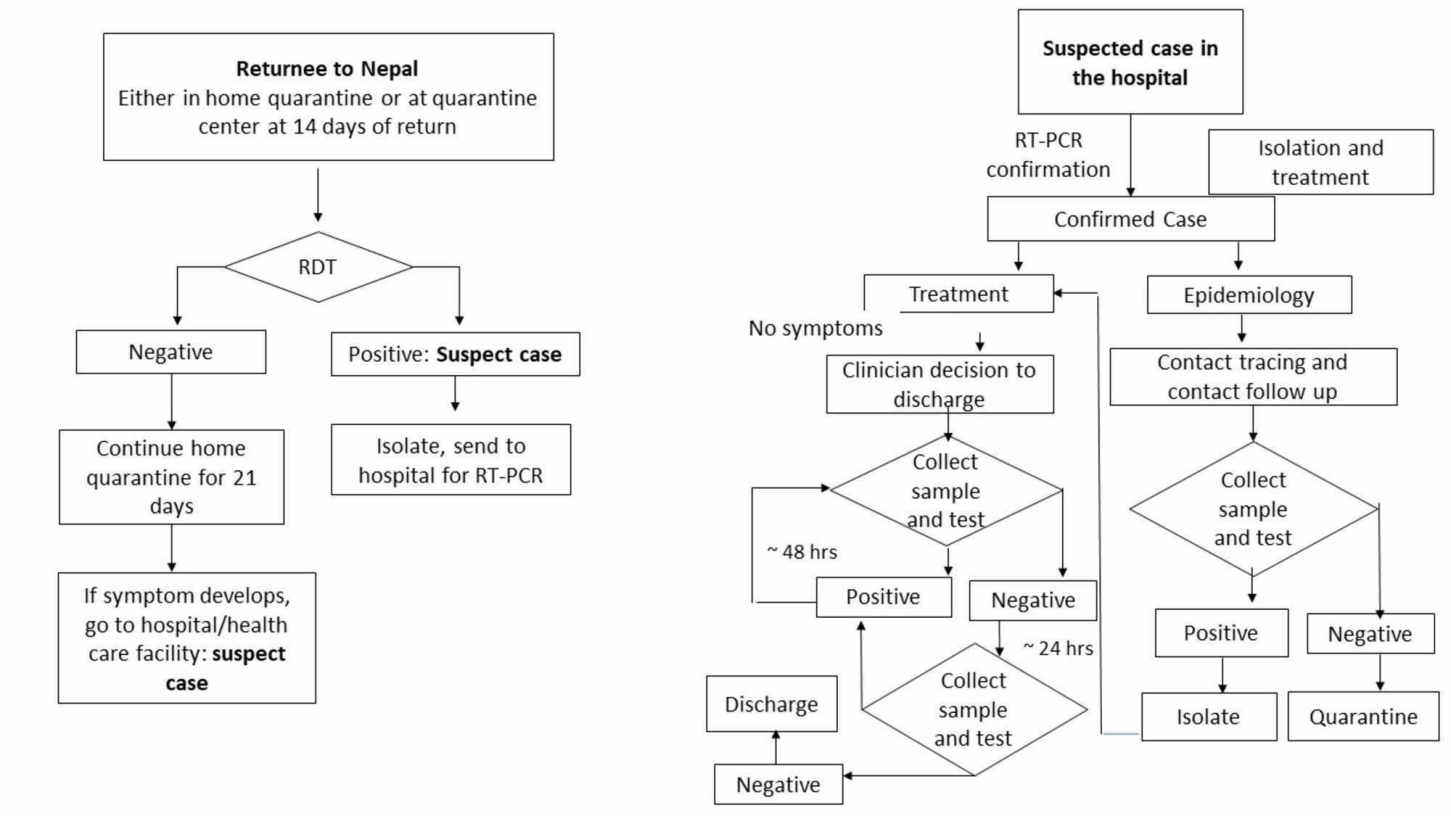
Protocols for individuals in quarantine and isolation Adapted from the Government of Nepal, Ministry of Health and Population. Health sector response to novel coronavirus. [8]

A rapid diagnostic test (RDT) for serology was initiated in Nepal in early April as a supplement to RT-PCR [9]. By mid-April, all districts throughout Nepal were equipped for RDT testing. This gave the opportunity for rapid, on-site tests for surveillance purposes that required minimum skill. Wide-spread testing was attempted on individuals entering Nepal (Figure 2).

**Figure 3:**
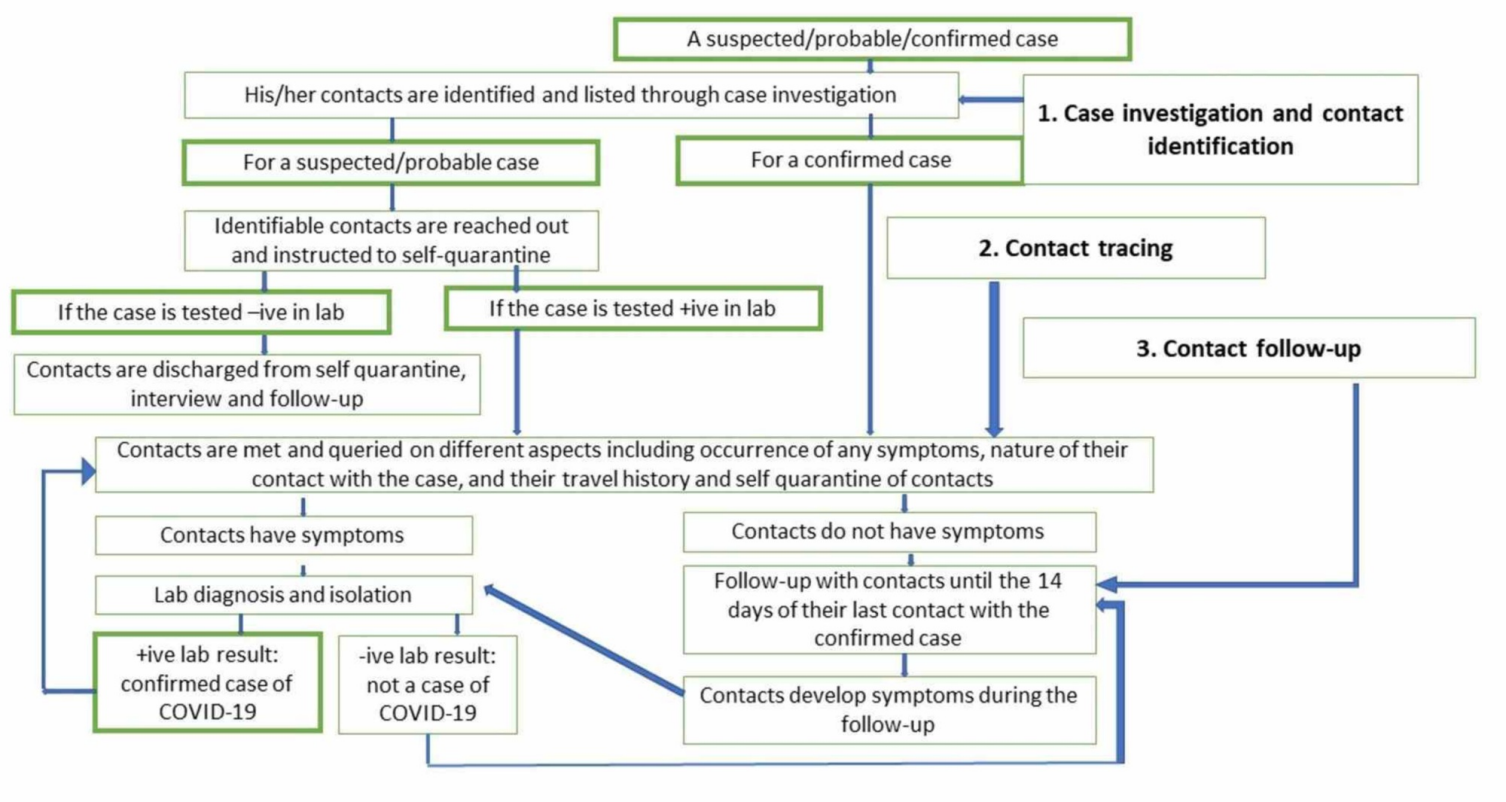
Steps: Case investigation and contact tracing after a case is suspected or confirmed Adapted from the Ministry of Health and Population. Epidemiology and Disease Control Division. Standard operating procedure for case investigation and contact tracing of COVID-19. Interim version. [14]

Figure 2 demonstrates algorithms for the testing protocol established on April 7, 2020, for all quarantined individuals and the mechanism for management and discharge once a suspect case was confirmed by RT-PCR [9]. Additionally, since RDT may not show the presence of positive antibodies in early stages and positive cases may be missed, if the cohort tested negative, 10% of the individuals in the cohort would receive PCR testing for surveillance. Positive cases were discharged after two samples of RT-PCR results negative 24 hours apart. Figure 3 elaborates the protocol for case investigation and contact identification of probable and suspect individuals, in addition to confirmed cases [15].

As of May 13, 21,340 RT-PCR assays and 60,319 RDT had been conducted with testing rates of 76 and 215 per 100,000 population, respectively [8,15]. On May 7, in order to increase the rate of testing in the limited number of facilities, PCR of pooled samples of individuals in quarantine and low risk for COVID-19 was started at a ratio of 1:5.

Risk Communication

Social media campaigns, including Viber, Facebook, websites, in addition to pamphlets, radio, and television focused on educating the public on strategies to prevent transmission [9]. Two toll-free call centers were established in order to provide counseling and information to citizens. A mobile application was set up for individuals to be able to assess their health status. If their self-assessment was concerning, health care workers from the Ministry of Health contacted them for the further need for testing or management. Daily briefings were broadcast by the health ministry via television and radio to share the current state to update the public and debunk false information.

Infected cases

**Figure 4:**
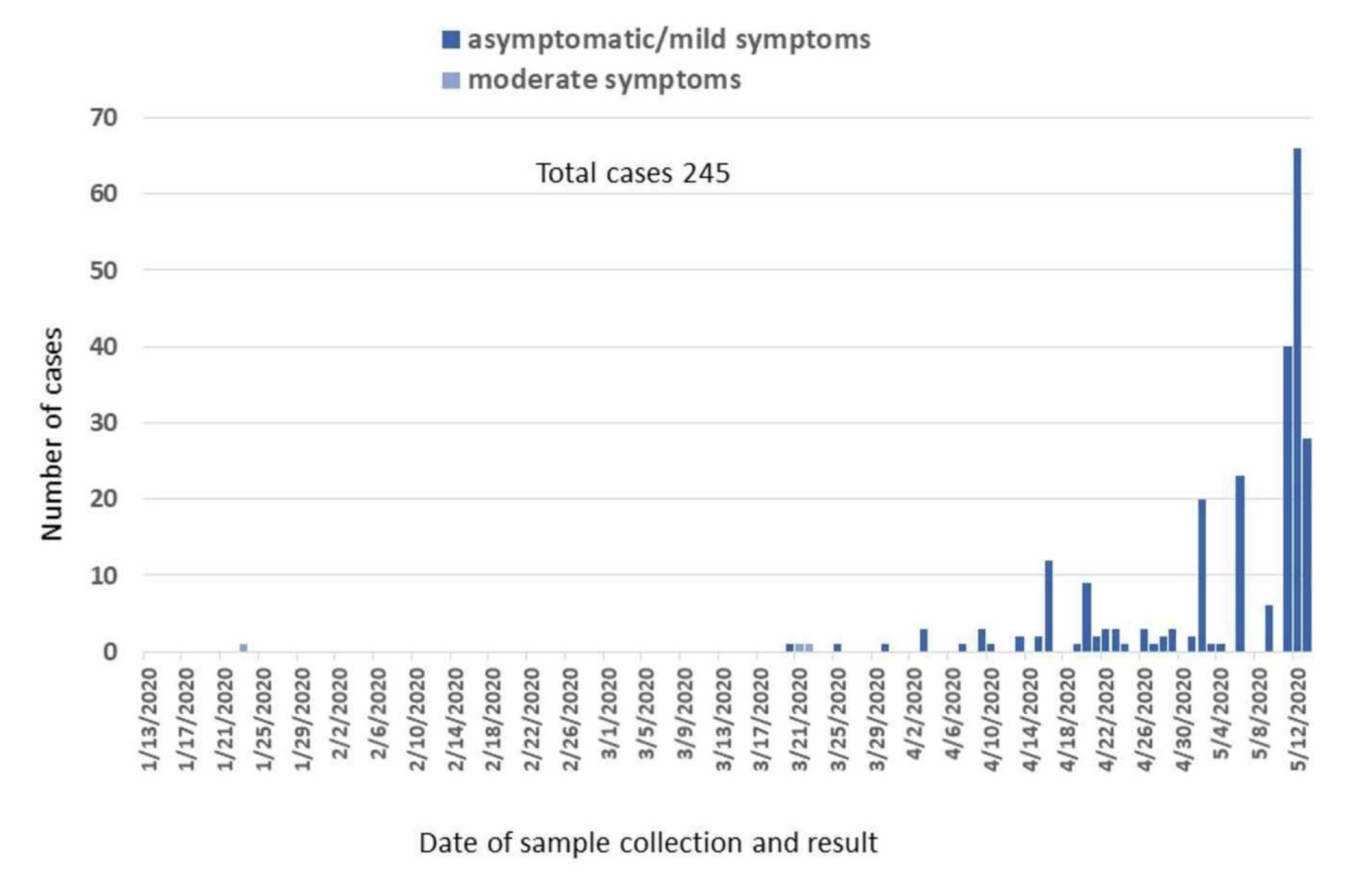
Epidemic curve of laboratory-confirmed COVID-19 cases by date of confirmation by RT-PCR, January 13, 2020, to May 13, 2020 RT-PCR: reverse transcription-polymerase chain reaction

After the initial cases introduced into Kathmandu by flight in early March as detailed above, there was a cohort of a few dozen Indian nationals adjacent to the border entering Nepal from India by land that tested positive. Early May, there was community transmission, resulting in a handful of SARS-CoV-2 positive cases in individuals who were living in Nepal. Then, the surge in cases in the second week of May occurred as a result of infected Nepali migrant workers returning home. Males between 18 and 45 years of age consisted of 80% of confirmed cases. As of May 13, all but 3 infected cases were asymptomatic or had mild symptoms, with no deaths or even intensive care admissions (Figure 4) [9].

Challenges

Rapid Rise in Confirmed Cases

The rate of infection in Nepal has been increasing exponentially. Just in the final three days of the four-month period of this review, confirmed cases doubled from 110 to 245 [9]. A little over a month later, right before the publication of this article, it has increased to over 10,000 with 24 deaths.

This exponential increase is as a result of an influx of infected cases crossing the open border between India and Nepal as the rate of infection in India escalated [16]. The economic upheaval caused by the shut-down in India forced thousands of Nepali migrant workers to attempt to enter Nepal [17-18]. Additionally, Indian nationals traveled into Nepal for religious, social, and trade reasons. There is no requirement for a visa or passport for citizens of these two nations to cross over. Most individuals enter Nepal via unguarded territories to avoid quarantine thus increasing the risk of community spread of the virus [19].

This influx has resulted in overwhelmed government-run quarantine and isolation facilities with shared confined spaces. This has, most likely, resulted in the in-facility transmission of the disease. Poverty makes home quarantine a poor alternative in a country like Nepal where large families live in crowded homes with scarce water and bathroom facilities. Ultimately, this can result in unchecked community transmission [19].

Inadequate Testing

Most individuals in quarantine are tested only at the end of two weeks with the serological method before being released to go home [9]. This increases the period of close contact. Inadequate resources and funding compounded by a lack of automated PCR machines limits prompt testing with RT-PCR, which could have led to earlier triage. A major barrier is Nepal’s dependence on external support and the import of essential health commodities for the COVID response, including the diagnostic tools (RT-PCR and serology). At the beginning of the outbreak, Nepal also lacked adequate molecular testing facilities and trained manpower except for a few places.

Health Infrastructure

Inadequate trained manpower and insufficient essential facilities will make it difficult to manage the surge of symptomatic and critical COVID-19 patients. There are 23 medical doctors per 10,000 population. Two-thirds are working in the few major cities of Nepal, including Kathmandu [20]. One recent study reported that medical doctors and nurses were physically present in only 18% and 41% of surveyed primary healthcare centers, respectively. Furthermore, 71% of staff consisted of semiskilled and unskilled workers [21]. Additionally, Nepal lacks enough hospital beds, intensive care units, ventilators, drugs, and necessary PPEs as well as expert manpower such as trained intensivists, respiratory therapists, skilled intensive care unit (ICU) nurses, and infectious disease and other sub-specialists [7].

Limited Surveillance

The country needs to expand its ability for the epidemiological surveillance system and research, both of which are in infantile form at present. Not enough field staff and community workers are included or trained in surveillance and contact tracing work. This can lead to difficulty in the identification of cases as the surge continues to escalate.

Social and Economic Upheaval

Similar to the rest of the world, Nepal is seeing more suffering, hunger, disease, and poverty as a result of the lockdown [22]. A report from John’s Hopkins Bloomberg School of Public Health projects up to 4000 under-five deaths in Nepal over just six months due to a reduction in the coverage of essential maternal and child health interventions, including family planning, antenatal and postnatal care, child delivery, vaccinations, food, water, and preventive and curative services [23-24]. Almost 40% of the population in the country, living below the $3.20 per person per day international poverty line, who are already at risk may be further pushed into extreme poverty as a result of COVID-19 [25]. Nepal is seeing a nationwide increase in suicide rates, by 20% according to some reports, attributed to the lockdown and poverty [26].

## Conclusions

Despite Nepal's poverty and lack of infrastructure, an early comprehensive COVID-19 preparedness plan was successful in deflecting the epidemic for the first few months. Effective measures included a strictly enforced lockdown, border control to prevent the importation of cases, mandatory institutionalized quarantine for all entering the country, identifying cases by implementing extensive contact tracing, and isolation of all cases irrespective of symptoms. For four months, starting with the first case on January 13 to May 13, 2020, these measures were very effective at flattening the curve. There were about 100 cases until early May without evidence of community spread, until the further entry of the virus via international travelers. The greatest limitation is the open border with India with thousands of migrant Nepali workers returning home. Right before this publication, there are almost a hundred-thousand individuals in cramped quarantine facilities, and 76 of the 77 districts are affected. However, most of these cases are being reported from quarantine facilities and community transmission has not been reported in most parts of the country so far. Continuing a strict lockdown may not be sustainable for the country anymore except in areas with evidence of community transmission. Better provisions for quarantine, ramping up RT-PCR services, increased surveillance, and contact tracing may mitigate some of the issues. Additionally, continued behavior interventions, such as social and physical distancing, the use of masks, and hand hygiene, must be encouraged.
